# Diagnostic Accuracy of Convolutional Neural Networks for Schizophrenia: A Systematic Review and Meta-Analysis.

**DOI:** 10.1192/j.eurpsy.2026.12168

**Published:** 2026-07-31

**Authors:** I. D. Hernández, E. Saucedo, A. Gogeascoechea, D. E. Guajardo, R. Huereca, A. L. Ponce, X. Triana, D. Morales

**Affiliations:** 1 Psychiatry Department, Hospital Universitario José Eleuterio Gonzalez; 2 Psychiatry Department, UANL Center for Advanced Neurosciences, Monterrey, Mexico

## Abstract

**Introduction:**

Schizophrenia remains a major diagnostic challenge due to its clinical heterogeneity and the lack of objective biomarkers. Deep learning (DL), particularly convolutional neural networks (CNNs), enables automated detection of complex neurobiological patterns from neuroimaging data. This study systematically reviewed and meta-analyzed the diagnostic accuracy of CNN-based models applied to electroencephalography (EEG), functional magnetic resonance imaging (fMRI), and structural MRI (sMRI) for schizophrenia classification.

**Objectives:**

To quantify pooled sensitivity and specificity of CNN-based deep learning models for schizophrenia diagnosis across EEG, fMRI, and sMRI modalities, and to evaluate heterogeneity and methodological quality among studies.

**Methods:**

A systematic search of six databases (Medline, Embase, Web of Science, Cochrane Central, PsycInfo, Scopus) was conducted in July 2024, following PRISMA 2020 guidelines (PROSPERO: CRD42024550691). Eligible studies applied CNN architectures to adult schizophrenia cohorts diagnosed per DSM or ICD criteria, reporting diagnostic accuracy metrics. Two reviewers independently extracted data and assessed quality using a modified QUADAS-2 tool tailored for AI/ML studies. Random-effects meta-analyses were performed to pool sensitivity and specificity; summary receiver operating characteristic (SROC) curves were generated for each modality.

**Results:**

Thirty-five studies (n≈13,100 participants) met inclusion criteria. CNN models achieved a pooled sensitivity of 0.90 [95% CI 0.85–0.94] and specificity of 0.90 [0.84–0.93]. EEG-based models reached the highest AUC (0.94) (Figure 1), followed by sMRI (0.91) (Figure 2) and fMRI (0.88) (Figure 3). Multimodal models combining imaging types outperformed unimodal approaches (AUC > 0.95). Moderate heterogeneity (I²≈40%) was observed across studies.

**Image 1:**
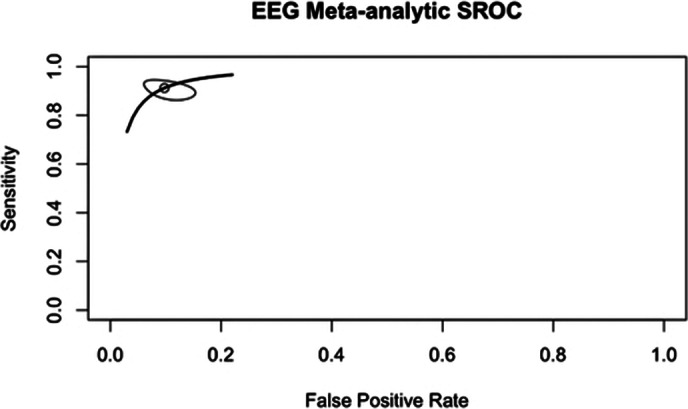

**Image 2:**
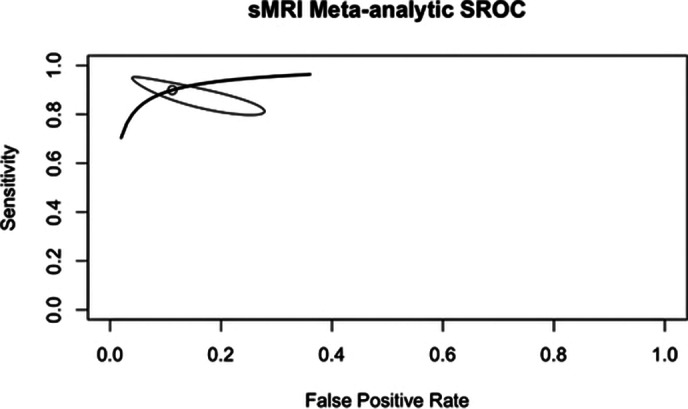
Image 2: Long description.

**Image 3:**
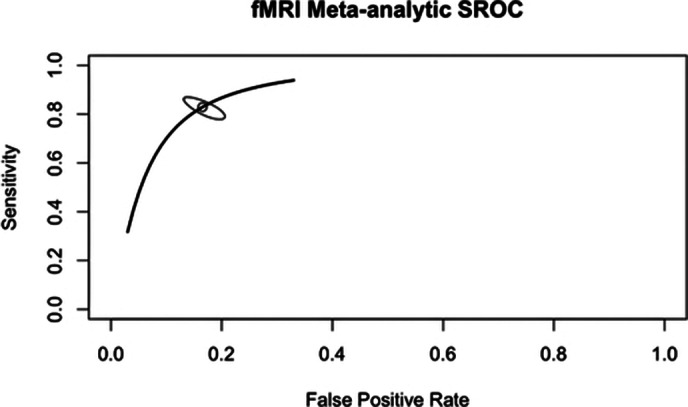
Image 3: Long description.

**Conclusions:**

CNN-based deep learning models show strong potential to assist schizophrenia diagnosis through neuroimaging and EEG data, achieving accuracies comparable to established clinical biomarkers. Standardization of preprocessing pipelines, external validation, and incorporation of explainable AI techniques are crucial to advance clinical translation.

**Disclosure of Interest:**

None Declared

## Long descriptions

### Image 2: Long description

The x-axis is labeled False Positive Rate with a scale from 0.0 to 1.0. The y-axis is labeled Sensitivity with a scale from 0.0 to 1.0. A thick black curve represents the S R O C line, which begins at approximately x = 0.02, y = 0.7 and follows an asymptotic curve upward and to the right, reaching y = 0.95 at x = 0.35. A thin black ellipse is centered at approximately x = 0.15, y = 0.9, oriented diagonally from the top-left to the bottom-right. A small open circle is plotted on the S R O C curve at the point where it intersects the upper-left boundary of the ellipse, at roughly x = 0.1, y = 0.9.

Navigate back to Image 2:.

### Image 3: Long description

The graph plots Sensitivity on the Y-axis from 0.0 to 1.0 against False Positive Rate on the X-axis from 0.0 to 1.0.

* A thick black curve represents the S R O C (Summary Receiver Operating Characteristic) line. It begins at approximately (0.05, 0.3) and rises steeply in a logarithmic-like curve, becoming more gradual as it approaches (0.35, 0.95).

* Located on the curve at roughly (0.18, 0.82) is a small open circle representing the summary point estimate.

* Surrounding this point are two concentric ellipses. The inner, smaller ellipse represents the 95 percent confidence region. The outer, larger and more elongated ellipse represents the 95 percent prediction region. Both ellipses are tilted diagonally from the bottom-left to the top-right, following the trajectory of the S R O C curve.

Navigate back to Image 3:.

